# Genome-Wide Identification, Phylogeny and Expression Analysis of Subtilisin (SBT) Gene Family under Wheat Biotic and Abiotic Stress

**DOI:** 10.3390/plants12173065

**Published:** 2023-08-25

**Authors:** Xiaotong Zhao, Farhan Goher, Lei Chen, Jiancheng Song, Jiqiang Zhao

**Affiliations:** 1 School of Life Science, Yantai University, Yantai 264005, China; zhaoxiaotong35910@163.com (X.Z.); chenlei119205@163.com (L.C.); jcsong88@163.com (J.S.); 2 State Key Laboratory of Crop Stress Biology for Arid Areas, College of Plant Protection, Northwest A&F University, Yangling 712100, China; goherfarhan@nwafu.edu.cn

**Keywords:** wheat, TaSBT family, stripe rust of wheat, biotic and abiotic stresses, expression analysis

## Abstract

The subtilisin-like protease (SBT) family is widely known for its role in stress resistance to a number of stressors in different plant species, but is rarely studied in wheat. Subtilisin-like serine proteases (SBTs) are serine proteolytic enzymes that hydrolyze proteins into small peptides, which bind to receptors as signal molecules or ligands and participate in signal transduction. In this study, we identified 255 putative SBT genes from the wheat reference genome and then divided these into seven clades. Subsequently, we performed syntenic relation analysis, exon-intron organization, motif composition, and *cis*-element analysis. Further, expression analysis based on RNA-seq and tissue-specific expression patterns revealed that *TaSBT* gene family expression has multiple intrinsic functions during various abiotic and biotic stresses. Analysis of RNA-seq expression assays and further validation through qRT PCR suggested that some of the *TaSBT* genes have significant changes in expression levels during *Pst* interaction. *TaSBT7*, *TaSBT26*, *TaSBT102*, and *TaSBT193* genes showed increasing expression levels during compatible and non-compatible interactions, while the expression levels of *TaSBT111* and *TaSBT213* showed a decreasing trend, indicating that these members of the wheat *SBT* gene family may have a role in wheat’s defense against pathogens. In conclusion, these results expand our understanding of the *SBT* gene family, and provide a valuable reference for future research on the stress resistance function and comprehensive data of wheat SBT members.

## 1. Introduction

Wheat (*Triticum aestivum*) is one of the important food crops, but in recent years, a variety of biological stresses have seriously affected its yield and quality. Wheat stripe rust, is one of the important wheat diseases, widely distributed around the world. Wheat stripe rust pathogen *Puccinia striiformis* f. sp. *tritici* is a basidiomycetous fungus [1] This disease may occur during the emergence of wheat to its maturity, and the main part that is damaged is the leaf. It has been reported that wheat stripe rust has a devastating effect on wheat production, which can lead to more than 40% yield reduction or no wheat harvest in pandemic years [2] Control of stripe rust currently includes chemical and biological control. However, a more economical and eco-friendly way to control *Pst* is to find a genetic source of resistance against the disease.

Subtilisin-like proteases (SBTs) are serine proteases with catalytic triplets of aspartic, histidine, and serine amino acids. Proteolysis enables amino acid cycling and facilitates post-translational modification [3]. In order to maintain accurate protein levels, a large number of proteases are widely distributed in plant cells. Among them, subtilisin (SBT) is the second largest member of the serine protease family, widely found in plants, fungi, bacteria, parasites, etc., and is related to plant growth and development and defense response. The SBT protein family has a highly conserved domain, peptidase_S8 (PF00082) [4]. which is used as a specific substrate binding site. In addition, the protease-related (PA) domain (PF02225) and the Inhibitor_I9 domain (PF05922) have been found among plants’ SBTs [5]. The SBT family is involved in a variety of cell activities and physiological processes; these proteases can catalyze protein, participate in autophagy processes, and are often expressed in plants under stress [6].

The SBT gene family was first identified in Cucumisin in melon [7], and later in tomato [8], grape [9], rubber [10], rice [11], poplar [12] and cotton [13]. A total of 56 SBT genes have been identified in *Arabidopsis thaliana*. In Arabidopsis, AIR3 (AtSBT5.3) promotes lateral root formation [14] and XSP1(AtSBT4.14) is involved in regulating xylem differentiation [15]. The protein encoded by GmSBT in soybean showed strict substrate specificity and induced the decomposition of soybean seed storage proteins. However, SCS1 (Subtilis subtilis 1) is preferentially expressed in soybean seeds and is involved in the remodeling of cell wall structures during seed coat development [12]. The expression of ApSBT in baizi lotus is responsive to osmosis, oxidation, and salt stress [16]. Loss of AtSBT3.3 function can impair innate immune functions, while overexpression of this gene can enhance plant resistance to pathogens [17]. At present, the identification of the wheat SBT family and the systematic description of their interactions with stripe rust fungus have not been studied.

In this study, starting from the genome-wide investigation of *TaSBT* genes, 255 putative *TaSBT* genes were identified through the whole-genome analysis of the TaSBT family, and were divided into 7 subgroups according to the phylogenetic relationship. Bioinformatics analysis was carried out to further study the exon-intron organization, motif structure, *cis*-acting elements arrangements, and synteny. In addition, expression profiling of *TaSBT* whole family during *Pst*-wheat interaction was also analyzed. This study could provide a reference for further study deep into the functional role of SBT family in other plant species, especially the wheat cereal relatives.

## 2. Results

### 2.1. Genome-Wide Identification of Wheat SBT Protein Family

The 255 *TaSBT* genes have been identified in the wheat genome were named according to chromosome localization. The length of TaSBT protein varied from 220 amino acids (TaSBT253) to 1491 amino acids (TaSBT235), and the corresponding molecular weight ranged from 24.22 to 163.20 Kd, indicating that there were some differences in molecular weight among members of the TaSBT family (Table S1). The isoelectric point (PI) ranges from 4.89 (TaSBT99) to 9.65 (TaSBT103), with equal amounts of acidic and basic amino acids, and 235 TaSBTs had plant-specific PA domains. In total, 218 TaSBTs possess a peptidase inhibitor I9 domain that plays a role in enzyme activation. Subcellular localization prediction analysis showed that TaSBTs were localized in chloroplasts (104), cell wall (58), vacuolar membrane (52), plasma membrane (21), endoplasmic reticulum (17), cytoplasm (15), and nucleus (8), with the largest number of loci in chloroplasts (Table S1).

### 2.2. Phylogenetic Analysis of Wheat SBTs

**Figure 1:**
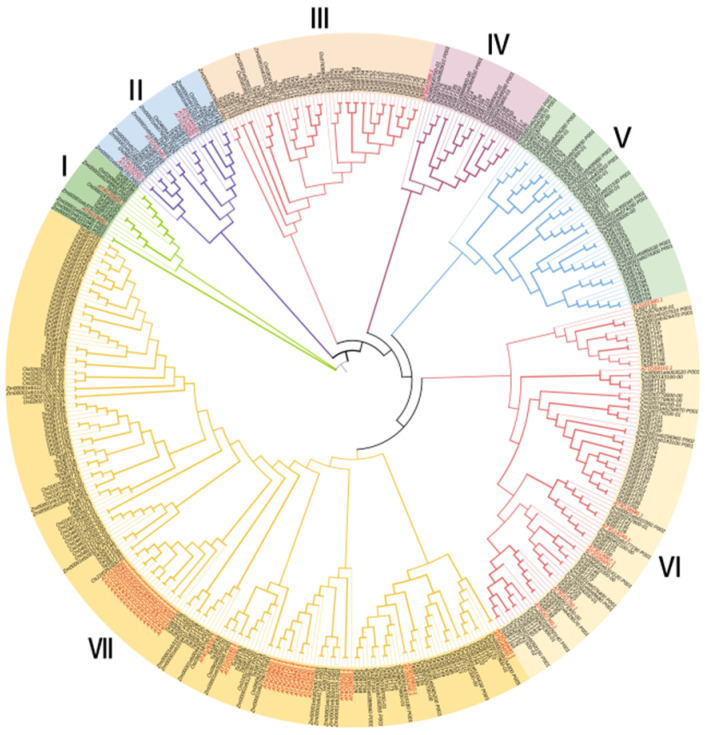
Phylogenetic tree of SBT gene using MEGA7, phylogenetic relationships of 438 SBT proteins in wheat, Arabidopsis, maize, and rice using neighbor linkage method.

In order to study the evolutionary relationship of SBT in wheat, *Arabidopsis thaliana*, rice, and maize, phylogenetic analysis was performed, with total of 438 SBT protein sequences (Figure 1), among which 255 were TaSBTs, 56 were AtSBTs, 66 were OsSBT66, and 61 were ZmSBTs. The phylogenetic tree showed that 438 members were divided into seven groups, Group I to Group Ⅶ. Group Ⅶ was the largest group, with 182 members, including TaSBTs (90), AtSBTs (39), OsSBTs (28), and ZmSBTs (22).

### 2.3. Collinearity and Chromosome Analysis of Wheat SBT Genes

**Figure 2:**
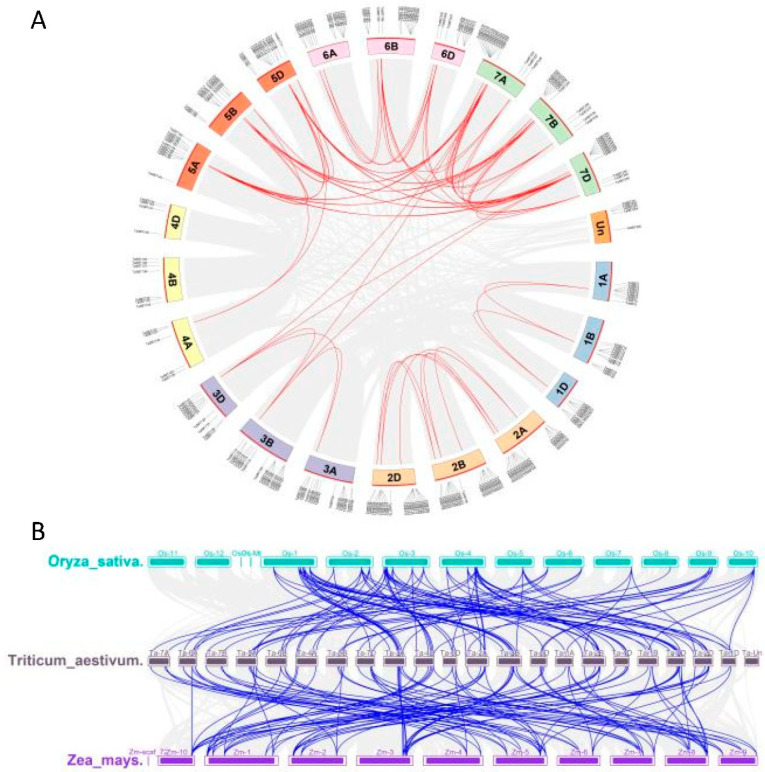
(**A**) Collinearity analysis of SBT genes in wheat, schematic diagram of chromosome distribution and relationship between chromosomes. (**B**) Collinear analysis of SBT genes in wheat, rice and maize. The red and blue lines highlight the SBTs with collinearity, and the gray lines represents the linear relationship of all genes.

Localization analysis of TaSBT chromosomes showed that 250 *TaSBT* genes were unevenly distributed on 21 chromosomes, and 5 genes had no loci. There were 86, 88, and 76 members from sub-genomes AA, BB, and DD, respectively, in wheat. In order to explore the possible mechanism of TaSBT amplification, we studied the gene replication events of wheat itself (Figure 2A). A total of 181 segment repeats and 74 single copy sequences were identified, indicating that segment repeats played a key role in the amplification of SBT in the wheat genome. In order to further explore the interspecies evolutionary mechanism of SBT family members, collinear analysis was conducted on SBTs of wheat, rice, and maize (Figure 2B). Results showed 81 homologous gene pairs in wheat and rice, and 90 gene pairs in wheat and maize, among which, 67 genes had collinear relationships among the three species, indicating that these genes were more conserved in evolution.

### 2.4. Gene structure Characterization and Protein Motif Analysis

**Figure 3:**
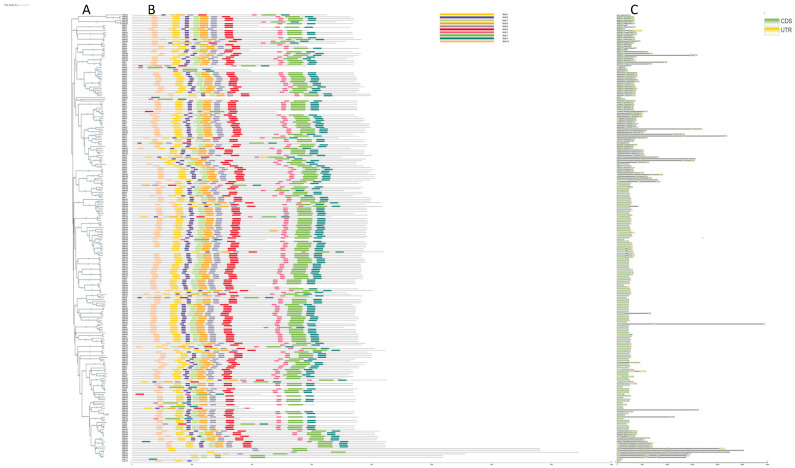
Gene structure and organization of conserved protein motifs. (**A**) The conserved motif composition of TaSBTs. Different motifs are displayed in boxes of different colors. (**B**) Exon-intron structure of TaSBTs. (**C**) The green box represents the CDS region, the yellow boxes represent the UTR region, and the black lines represent the untranslated intron.

In order to analyze the evolutionary relationship of TaSBT family members, an evolutionary tree of the *TaSBT* gene (Figure 3A) was constructed using the NJ method. The genes can be divided into 7 groups (Ⅰ~Ⅶ), which is consistent with the evolutionary analysis results of gene family (Figure 1). The motif structure has a specific function in protein molecules; an identified motif structure can describe the characteristics of this gene family. Most TaSBTs contained 10 motifs (Figure 3B), proving their strong conservatism. TaSBTs with closer topological structure groups in the evolutionary tree have similar feature domains. Genes structures were simple and conserved, with similar gene structures appearing in the same group, and the *TaSBT* gene with only one exon comes from Group VII (Figure 3C).

### 2.5. Identification of Cis-Acting Elements in TaSBTs Promoter Region

**Figure 4:**
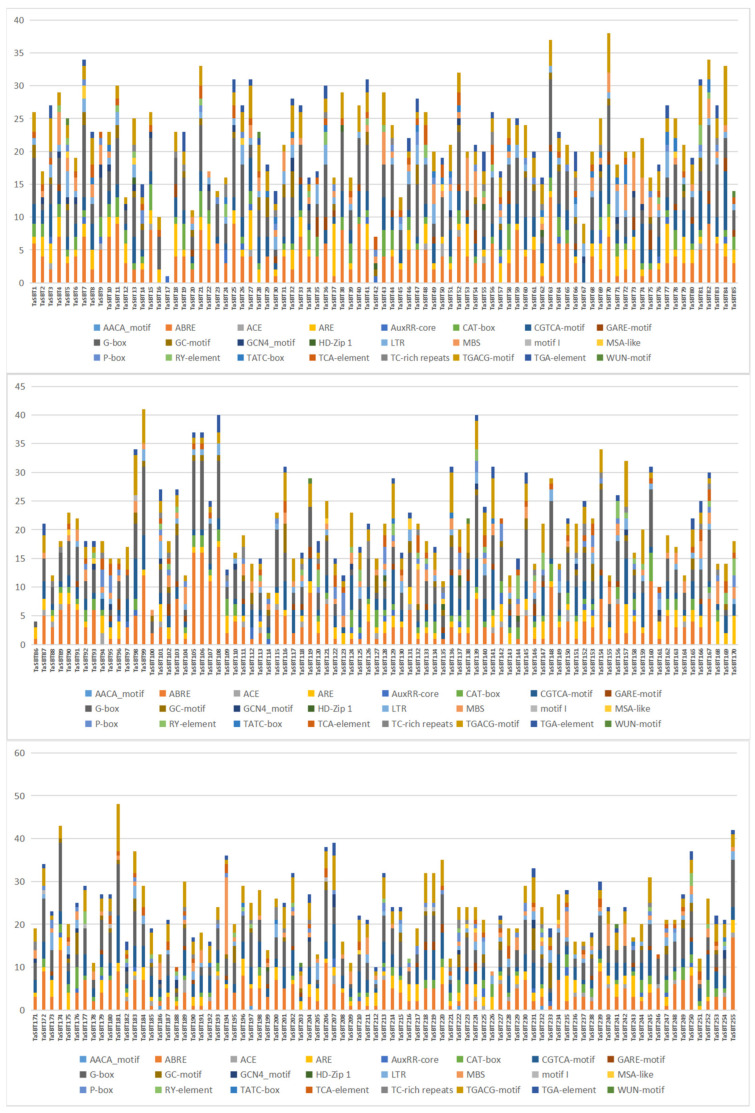
*Cis* acting elements in the promoter region of TaSBTs. The distribution, classification and proportion of *cis* acting elements in each gene promoter region.

Analysis of *cis*-acting elements in the promoter regions can reveal whether genes respond to hormones and stress, etc. A total of 24 kinds of *cis*-acting elements were identified in 255 TaSBT promoter regions, which were divided into three categories: growth-, stress-, and hormone-related (Figure 4). There are 9 *cis*-acting elements (AACA_motif, ACE, CAT box, G-box, GCN4_motif, HD-Zip1, motifI, MSA-like, RY element). In this category, the largest number of G-boxes were related to light (68.51%); 19.73% of the identified elements were classified as response elements related to stressors, which includes anaerobic induction (ARE, 36.01%), drought induction (MBS, 24.84%), low temperature (LTR, 19.21%), anoxia (GC motif, 13.85%), defense and stress (TC-rich repeats, 5.45%) and injury responsive (WUN motif, 0.63%). Among the hormones, 52.28% include abscisic acid (ABRE, 34.42%), methyl jasmonate (TGACG motif, 23.23%, CGTCA motif, 23.16%), gibberellin (P-box, 3.88%, GARE motif, 2.97%, TATC box, 1.18%), auxin (TGA element, 4.82%, AuxRR core, 1.72%), and salicylic acid (TCA element, 4.62%). TaSBT181 has 48 *cis*-acting elements, which is the most *cis*-acting gene, including 12 G-box, 11 CGTCA-motifs, 11 TGACG-motifs and 9 ABRE, indicating that TaSBT181 has potential functions in light response and plant hormones. TaSBT174 contains 43 *cis*-acting elements, mainly composed of 16 G-boxes, 14 ABRE, 3 CGTCA motifs, and 3 TGACG motifs, suggesting that TaSBT174 may play a role in light response and plant hormone response. It was speculated that TaSBT gene family might be closely related to light response, growth and development, hormone induction and biological stress of wheat.

### 2.6. Expression Pattern of SBT Genes in Wheat

**Figure 5:**
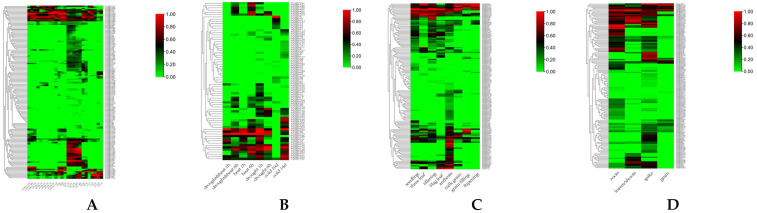
(**A**) Differential expression heat map of TaSBTs in response to five biological stresses, including powdery mildew (P), stripe rust (CYR31, 87/66), scab (FG), stem base rot (FP), and wheat leaf blight (Z). (**B**) Differential expression heat map of TaSBTs in response to four abiotic stresses, including drought & heat, heat, drought, and cold treatments. (**C**) Heat map of TaSBT differential expression related to wheat growth and developmental stages, including seedling, three leaf, tillering, flag leaf, anthesis, milk grain, grain filling, and ripening. (**D**) Expression profile of TaSBTs in four tissues of wheat.

The response of *TaSBT* genes to biological stress was assessed by RNA-seq data. Results showed 64 genes expressed during exposure to stripe rust (*Puccinia striiformis* f. sp. *tritici*) interaction, 54 genes were up-regulated during exposure to powdery mildew (*Blumeria graminis*), 116 genes were up-regulated during exposure to *Fusarium graminearum*, 75 were up-regulated were during *Fusarium pseudograminearum* infection, and 43 were up-regulated during exposure to wheat leaf blight (*Zymoseptoria tritici*) infection (Figure 5A). The expression levels of six genes, *TaSBT7*, *TaSBT26*, *TaSBT102*, *TaSBT111*, *TaSBT193*, and *TaSBT213*, showed significant induction upon bacterial inoculation, and were preliminarily selected for future research. Numerous gene expression levels were significantly up-regulated after inoculation with stripe rust (12), powdery mildew (17), wheat scab (31), stem rot (17) and wheat leaf blight (21), indicating that they may likely to be involved in the regulation of corresponding diseases. In addition to biological stress, wheat also faced a variety of abiotic stresses. The expression pattern of the TaSBT family under abiotic stress was also analyzed (Figure 5B). Under drought stress, 47 *TaSBT*s showed differential expression, and the expression levels of 10 genes significantly increased, while the expression levels of most *TaSBT*s decreased with the extension of drought treatment. In addition, 37 *TASBT*s responded to heat stress, and 9 of these were up-regulated. Under combined stress of drought and heat, 34 genes were differentially expressed, and 6 genes were significantly up-regulated. In response to cold stress, 49 *TaSBT*s were differentially expressed and 7 were up-regulated. A total of 140 *TaSBT* genes were differentially expressed during the growth and development of wheat. There were many differentially expressed genes during 8 stages of the growth period (Figure 5C), at the seedling stage (77), tri-lophyll stage (59), tiller stage (73), flag leaf stage (63), flowering stage (110), milk ripening stage (42), grain filling stage (29), and maturity stage (28). Overall, there were different up-regulated genes at each stage, and the largest number of genes were up-regulated at seedling stage. By analyzing the differential expression of *TaSBTs* in different tissues of wheat, it was found that 93, 54, 110, and 30 genes among 142 genes were differentially expressed in root, leaf, ear, and seed, respectively, suggesting that *TaSBTs* mainly played a role in wheat tissue expression (Figure 5D). The expression levels of *TaSBT105* in root, *TaSBT197* in leaf, *TaSBT27* in ear, and *TaSBT154* in seed were significantly up-regulated, which might be related to their expression, providing reference for subsequent research.

### 2.7. GO Enrichment Analysis of Differentially Expressed Genes

**Figure 6:**
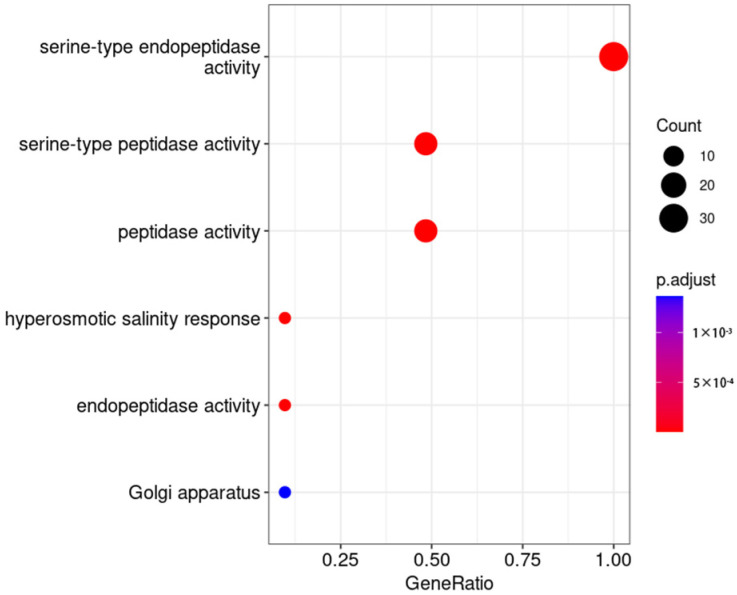
GO enrichment analysis of TaSBTs related to stripe rust stress. Each row corresponds to a valid GO item; The list shows GeneRatio (genes enriched in differentially expressed genes in the pathway/functional genes in differentially expressed genes). The bubble size represents the number of genes, and the color gradient represents—log10 (*p*-value).

In order to explore the regulatory pathways of these TaSBTs, GO enrichment analysis was conducted on wheat materials during the seedling stage (Figure 6). We have identified 63 unique GO terms for biological processes (BP), molecular functions (MF), and cellular components (CC) (Table S2, Figure 6). We found that TaSBTs are mainly related to the regulatory process of serine peptidases, such as serine-type endopeptidase activity, serine-type peptidase activity, and endopeptidase activity. Interestingly, we have identified several immune-related GO terms, including cellular signaling, gibberellic acid mediated signaling pathways, ubiquitin-plasma system control of cycloplasmic protein quality, xylan acetylation, and UDP-L-arabinose metabolism processes.

### 2.8. qRT-PCR Expression Analysis of TaSBTs during Pst Interaction

**Figure 7:**
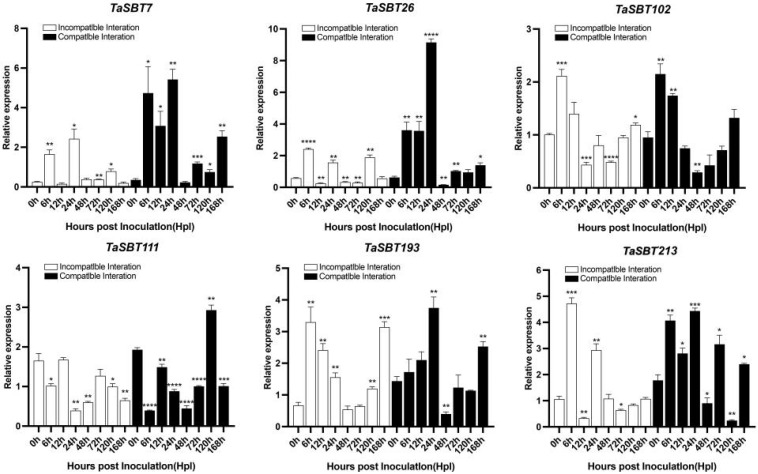
Expression analysis of *TaSBT7*, *TaSBT26*, *TaSBT102*, *TaSBT111*, *TaSBT193*, and *TaSBT213* under stripe rust infection. The error bars in the figure indicate standard errors, while asterisks indicate significant differences (* *p* < 0.05, ** *p* < 0.01, *** *p* < 0.001, **** *p* < 0.0001).

To further validate the expression of these six genes that showed significant expression levels in RNA seq analysis, we conducted qRT-PCR expression assays (Figure 7). The results showed that under the incompatible (CYR23) interaction, *TaSBT7* was significantly upregulated at early stages of interaction, at 6 and 24 hpi (hours post inoculation), *TaSBT26* showed strong induction of expression and peaked at 24 hpi, during the compatible interaction (CYR31). *TaSBT102* at 6 hpi, displayed higher induction of transcripts level, while *TaSBT111* showed strong significant expression at a later stage of interaction, at 120 hpi during compatible interaction compared to mock. Under the incompatibile (CYR23) interaction, *TaSBT193* and *TaSBT213* showed significant induction of expression at an early stage of interaction, 6 hpi. Data suggests that these TaSBT family members may have a role during wheat defense against *Pst*.

## 3. Materials and Methods

### 3.1. RNA Extraction, Stripe Rust Inoculation and Plant Materials

The plant immunity research team of Northwest A&F University provided the stripe rust strain CYR31 or CYR23 and Mingxian 169 wheat materials. The seedlings were cultured under 22 °C and 16/8 h light/dark cycle conditions, up to the two leaves stage. The second leaf of the seedlings was inoculated with CYR31. After inoculation, the seedlings were placed in a dark environment at 6–12 °C and full moisture for 48 h, and then moved to 16 °C for normal growth up to the visible sporulation. The wheat cultivar Suwon 11 was infected with *Pst* race CYR23 (avirulent) or CYR31 (virulent). *Pst-*inoculated samples were collected at 0, 6, 12, 24, 48, 72, and 120 hpi [18], and immediately put in liquid nitrogen and kept at –80 °C until RNA extraction was performed.

### 3.2. Identification and Classification of Wheat TaSBT Gene Family

Wheat genome information was retrieved from the Ensembl Plants database [19]. PFAM [20] (https://pfam.xfam.org/; accessed on 5 January 2022) and the SBT domain (PF00082) [21] was obtained using the hidden Markov model (HMM) used in the HMMER3.0 [22] software. From the wheat-specific SBT-HMM model, SBTs with *E*-value < 0.001 were selected, using the HMM tool to search all possible SBT gene sequences in the wheat genome database. Candidate gene family members were selected from the NCBI-CDD (https://www.ncbi.nlm.nih.gov/cdd/; accessed on 7 January 2022) [23], using the reference domain to ensure that all identified sequences contain the target domain [24]. The ExPASyprotParam [25] online tool was used to obtain the identified physicochemical properties of TaSBT family members. WoLFPSORT [26] was used to perform subcellular localization prediction.

### 3.3. Structure, and Phylogenetic Analysis of Wheat SBT Proteins

The MEME database (https://meme-suite.org/meme/; accessed on 15 February 2022) [27] was used to submit TaSBT protein sequences to obtain the meme file, and the maximum number of motifs searched was 10 [28]. The Ensembl Plants database was used to obtain the whole genome and protein sequences of wheat (*Triticum aestivum*), *Arabidopsis thaliana*, rice (*Oryza sativa*), and maize (*Zea mays*) using the HMM-SBT model. All the retrieved sequences were submitted into MEGA7.0 [29] to construct the SBT phylogenetic tree, using the neighbor-joining method [30]. The saved raw data was processed using the iTOL online software (https://itol.embl.de/index.shtml) to further improve the phylogenetic tree (accessed on 12 January 2022) [31]. Genomic sequences of wheat, rice, and mazie, collected from the Ensembl Plants database and the collected location information of all identified wheat *SBT*s genes, including the starting position on the chromosome, localized chromosome number, and chromosome length. TBtools [32] software was used to analyze and visualize the collinearity relationship between wheat and the other three species.

### 3.4. Analysis of TaSBTs Gene Expression Profile and Cis-Acting Elements

RNA-Seq data were obtained from the Wheat Expression Browser (http://www.wheat-expression.com/; accessed on 26 February 2022) [33] to analyze the expression patterns of *TaSBT* genes under different conditions. These conditions include various biological and abiotic stresses, different growth and development stages in various plant tissues. The upstream 2000 bp sequence of *TaSBTs* family genes were extracted, and PlantCARE [34] was used to analyze the homeo dynamics of *cis*-elements in the promoter region. The annotated information provided by the IWGSC Reference Genome (IWGSC RefSeq v1.1) was used as a reference database for analyzing GO and KEGG data. Images were drawn using the R package clusterProfiler.

### 3.5. Expression Analysis of TaSBTs under Pst-Wheat Interaction

Total RNA was extracted with the Trizol reagent according to the manufacturer’s protocols (Invitrogen, Waltham, MA, USA), and then treated with DNase I (Promega, Madison, WI, USA) to remove DNA impurities. cDNA were then synthesized with GoScript Reverse Transcription System (Promega, USA) and an oligo (dT18) primer (Invitrogen, USA). Specific pairs of primers were used, including TaActin [35] as internal reference gene to quantify the expression level via qRT-PCR using the synthesized cDNA (Table S3). A 7500 Real-Time PCR System (Applied Biosystems, Carlsbad, CA, USA) was used to quantify the transcripts. The relative expression of *TaSBT*s was determined with comparative method 2^−ΔΔCT^ [36]. qRT-PCR experiments were carried out three times.

## 4. Discussion

Subtilisin is a protein digestive enzyme originally obtained from *Bacillus subtilis*. The mature form is a globular protein containing 275 residues, with several α spirals and a large β folding sheet. The N-terminal contains an I9 pre-peptide domain (InterPro: IPR010259), which facilitates the folding of subtilisin. Most proteases in plants are of the catalytic serine peptidase type. Among serine peptidases, those related to bacterial subtilisin constitute the largest family, so it is very important to study the role of the SBT gene family in regulation of plant growth and development, response to environmental regulation, and response to biotic stress. Its involvement in various cellular processes such as protein activation in many plants has been reported. This unique region is used as a binding site for specific substrates. The *SBT* gene family exists throughout the plant kingdom and plays a variety of roles in plant growth and defense [5]. The SBT family has been extensively studied in *Arabidopsis thaliana*, and a total of 56 AtSBT family members have been identified [37].

It has been reported that SBT protein exists widely in plants, fungi, bacteria, parasites, etc., and is relatively conserved among different plant species [6]. In this study, 255 *TaSBT* genes were identified from the wheat reference genome based on an HMM model. In order to study the phylogenetic relationships of wheat SBT genes, a phylogenetic tree was constructed. According to the phylogenetic relationships, wheat SBT members were divided into 7 groups (Figure 1). Studies of gene repetition events illustrate the potential spread mechanism of TaSBT, suggesting that duplicate gene pairs tend to come from the same subfamily (Figure 2). Gene structure and motif composition further demonstrated the relative conservatism among members of the same subfamily (Figure 3), and these results were consistent with previous studies [21].

*Cis*-acting element analysis showed that TaSBTs may be involved in light response, anaerobic induction, and biological stress. It has previously been reported that some SBT members are involved in plant growth and development, including embryo development, stomatal density regulation, and reproductive development. For example, in Arabidopsis and nightshade plants, stomatal development is inhibited through the TMM (TooManymouth)-dependent pathway [38]. In *Arabidopsis thaliana*, *AtSBT1.4* was expressed in all above-ground organs and down-regulated the seed setting rate and number of branched inflorescences during reproductive development [37]. AtSBT1.7 triggers the accumulation and/or activation of cell-wall-modifying enzymes that are required for external primary cell wall loosening or mucus expansion promotion, as indicated by increased pectin methyl esterase activity during AtSBT1.7 mutant seed development. In legumes (*Medicago truncatula* and *Pisum sativum*), SBT1.1 protein is located in the endosperm and controls changes in seed size by regulating embryonic cell division during reproductive development [37]. Along with the developmental role of the SBT family, various studies have also reported their role under stress. Chen Fajing et al. found that the *TaSBT1* gene affects plant disease resistance by regulating the activity of PME (Pectin methylesterase) in healthy plants, and in plants activated by depression of *TaSBT1*. Overexpression of the *AtSBT3.3* gene can enhance plant resistance to pathogens. It is worth noting, no *TaSBT* gene has been previously reported to have a role in wheat resistance against stripe rust fungus. Gene expression triggered by environmental stresses not only protects cells from injury, but also controls the expression of genes involved in signal transduction processes. Upon RNA-seq data analysis, here we also found six members of TaSBT family induced expression during biotic stress. Furthermore, the expression of these six genes, *TaSBT7*, *TaSBT26*, *TaSBT102*, *TaSBT111*, *TaSBT193*, and *TaSBT213* was validated through qRT-PCR analysis, and this showed significant up-regulation of expression during both compatible (CYR31) and incompatible interactions (CYR23). Thus, this suggests a possible role in wheat resistance against pathogen stress. In this study, several *TaSBT* candidate genes that may be involved in stripe rust interaction were identified. Future studies need to further examine the molecular mechanism of these screened TaSBT candidates during wheat and stripe rust interaction.

## 5. Conclusions

This study provides a genomic framework for the wheat *SBT* gene family and its phylogenetic relations with *Arabidopsis*, rice, and maize. A total of 255 *TaSBT* genes were identified from the wheat genome (BB, AA, DD). Extensive bioinformatics analysis provides a base for further study of this gene family in wheat. Analysis of spatial expression patterns suggests that the *SBT* gene family is probably involved in the response to stress conditions in wheat. This study provides a basis for understanding the biological functions of the *SBT* family genes in wheat during biotic and abiotic stresses. It also provides a reference for breeders, that offers candidate genes related to pathogen resistance for the achievement of sustainable production goals in the future.

## Data Availability

Not applicable.

## Supplementary Materials

The following supporting information can be downloaded at: https://www.mdpi.com/article/10.3390/plants12173065/s1, Table S1: Physico chemical properties of wheat SBT gene family; Table S2: GO enrichment between TaSBTs related to rust stress; Table S3: Primers for TaSBT7, 26, 102, 111, 193, 213 gene cloning.

## References

[1] Yi J.P., He J., Shan C.J. (2012). Principles and Diagnosis and Treatment of Common Plant Diseases.

[2] Yuan L.Y., Xie Y.J., Ye H.G. (2012). The occurrence and control of wheat stripe rust. Mod. Agric. Sci. Technol..

[3] Havé M., Balliau T., Cottyn-Boitte B., Dérond E., Cueff G., Soulay F., Lornac A., Reichman P., Dissmeyer N., Avice J.C. (2017). Increase of proteasome and papain-like cysteine protease activities in autophagy mutants: Backup compensatory effect or pro cell-death effect?. Exp. Bot..

[4] Chen F.J., Tan R., Huang M.Y., Du J., Yu Y., Yang Y.H., Bi C.W. (2018). Research progress on the interaction between plants and pathogens using subtilisin like proteases. Mol. Plant Breed..

[5] Schaller A., Stintzi A., Rivas S., Serrano I., Vera P. (2017). From structure to function—A family portrait of plant subtilases. New Phytol..

[6] Liao S.H. (2011). The biological function of Bacillus subtilis like protease and its application in disease control in agriculture and animal husbandry. Anhui Agric. Sci..

[7] Yamagata H., Masuzawa T., Nagaoka Y., Ohnishi T., Iwasaki T. (1994). Cucumisin, a serine protease from melon fruits, shares structural homology with subtilisin and is generated from a large precursor. Biol. Chem..

[8] Tornero P., Conejero V., Vera P. (1996). Primary structure and expression of a pathogen-induced protease (PR-P69) in tomato plants: Similarity of functional domains to subtilisin-like endoproteases. Proc. Natl. Acad. Sci. USA.

[9] Cao J., Han X., Zhang T., Yang Y., Huang J., Hu X. (2014). Genome-wide and molecular evolution analysis of the subtilase gene family in Vitis vinifera. BMC Genom..

[10] Ekchaweng K., Khunjan U., Churngchow N. (2017). Molecular cloning and characterization of three novel subtilisin-like serine protease genes from Hevea brasiliensis. Physiol. Mol. Plant Pathol..

[11] Tripathi L.P., Sowdhamini R. (2006). Cross genome comparisons of serine proteases in Arabidopsis and rice. BMC Genom..

[12] Schaller A., Stintzi A., Graff L. (2012). Subtilases—Versatile tools for protein turnover, plant development, and interactions with the environment. Physiol. Plant.

[13] Dai M., Zhou N., Zhang Y., Zhang Y., Ni K., Wu Z., Liu L., Wang X., Chen Q. (2023). Genome-wide analysis of the SBT gene family involved in drought tolerance in cotton. Front. Plant Sci..

[14] Neuteboom L.W., Veth-Tello L.M., Clijdesdale O.R., Hooykaas P.J.J., van der Zaal B.J. (1999). A Novel Subtilisin-like Protease Gene from Arabidopsis thaliana is Expressed at Sites of Lateral Root Emergence. DNA Res..

[15] Zhao C. (2000). Exploiting Secondary Growth in Arabidopsis. Construction of Xylem and Bark cDNA Libraries and Cloning of Three Xylem Endopeptidases. Plant Physiol..

[16] Liu P.L., Chen G.Q., Shen X.H. (2020). Cloning and Expression Characteristics Analysis of the ApSBT Gene of Bacillus subtilis Protease from Baizilian. Plant Physiol..

[17] Ramírez V., López A., Mauch-Mani B., Gil M., Vera P. (2013). An extracellular subtilase switch for immune priming in Arabidopsis. PLoS Pathog..

[18] Bai X.X., Zhan G.M., Tian S.X., Peng H., Cui X.Y., Islam M.A., Farhan G., Ma Y.Z., Kang Z.S., Xu Z.S. (2021). Transcription factor BZR2 activates chitinase Cht20. 2 transcription to confer resistance to wheat stripe rust. Plant Physiol..

[19] Bolser D.M., Kerhornou A., Walts B., Kersey P. (2015). Editor’s Choice: Triticeae Resources in Ensembl Plants. Plant Cell Physiol..

[20] Finn R.D., Tate J., Mistry J., Coggill P.C., Sammut S.J.J., Hotz H.R., Ceric G., Forslund K., Eddy S.R., Sonnhammer E.L. (2008). The Pfam protein families database. Nucleic Acids Res..

[21] Wen S., Chen G., Huang T., Shen X. (2020). Genome-wide Study of SBT Genes in Eight Plant Species: Evolution and Expression Outlines for Development and Stress. Res. Sq..

[22] Potter S.C., Luciani A., Eddy S.R., Park Y., Lopez R., Finn R.D. (2018). HMMER web server: 2018 update. Nucleic Acids Res..

[23] Lu S., Wang J., Chitsaz F., Derbyshire M.K., Geer R.C., Gonzales N.R., Gwadz M., Hurwitz D.I., Marchler G.H., Song J.S. (2020). CDD/SPARCLE: The conserved domain database in 2020. Nucleic Acids Res..

[24] Aron M.B., Lu S., Anderson J.B., Farideh C., Derbyshire M.K., DeWeese-Scott C., Fong J.H., Geer L.F., Geer R.C., Gonzales N.R. (2011). CDD: A Conserved Domain Database for the functional annotation of proteins. Nucleic Acids Res..

[25] Wilkins M.R., Gasteiger E., Bairoch A., Sanchez J.C., Hochstrasser D.F. (1999). Protein Identification and Analysis Tools in the ExPASy Server. Methods Mol. Biol..

[26] Horton P., Park K.J., Obayashi T., Harada H., Adams-Collier C.J., Nakai K. (2007). WoLF PSORT: Protein Localization Prediction Software. Nucleic Acids Res..

[27] Bailey T.L., Johnson J., Grant C.E., Noble W.S. (2015). The MEME Suite. Nucleic Acids Res..

[28] Granziol D., Ru B., Zohren S., Dong X., Roberts S. (2019). MEMe: An Accurate Maximum Entropy Method for Efficient Approximations in Large-Scale Machine Learning. Entropy.

[29] Sudhir K., Glen S., Koichiro T. (2016). MEGA7: Molecular Evolutionary Genetics Analysis Version 7.0 for Bigger Datasets. Mol. Biol. Evol..

[30] Guo B.J., Li Y., Yuan Z.C., Lu C., Zhang X.Z., Xu R.G. (2016). Genome wide analysis of barley ARF gene family. Wheat Crops.

[31] Letunic I., Bork P. (1988). Interactive Tree of Life (iTOL): An online tool for phylogenetic tree display and annotation. FEBS Lett..

[32] Chen C., Chen H., Zhang Y., Thomas H.R., Frank M.H., He Y.J., Xia R. (2020). TBtools: An Integrative Toolkit Developed for Interactive Analyses of Big Biological Data. Mol. Plant.

[33] Ramírez-González R., Borrill P., Lang D., Harrington S., Brinton J., Venturini L., Davey M., Jacobs J., Van E.F., Pasha A. (2018). The transcriptional landscape of polyploid wheat. Science.

[34] Lescot M. (2002). PlantCARE, a database of plant cis-acting regulatory elements and a portal to tools for in silico analysis of promoter sequences. Nucleic Acids Res..

[35] Dominguez R., Holmes K.C. (2011). Actin structure and function. Annu. Rev. Biophys..

[36] Livak K.J., Thomas D.S. (2001). Analysis of relative gene expression data using real-time quantitative PCR and the 2^−ΔΔCT^ method. Methods.

[37] Rautengarten C., Steinhauser D., Büssis D., Stintzi A., Schaller A., Kopka J., Altmann T. (2005). Inferring Hypotheses on Functional Relationships of Genes: Analysis of the Arabidopsis thaliana Subtilase Gene Family. PLoS Comput. Biol..

[38] Wang G., Ellendorff U., Kemp B., Mansfield J.W., Forsyth A., Mitchell K., Bastas K., Liu C.M., Woods-Tör A., Zipfel C. (2008). A genome-wide functional investigation into the roles of receptor-like proteins in Arabidopsis. Plant Physiol..

