# Supplementary material for: Genome-Wide Identification, Phylogeny and Expression Analysis of Subtilisin (SBT) Gene Family under Wheat Biotic and Abiotic Stress
Source: Plants (Basel). 2023 Aug 25;12(17):3065. doi: 10.3390/plants12173065 (PMC10489890; doi:10.3390/plants12173065)
Supplement: Supplementary file 1 [file plants-12-03065-s001.zip › Table S1.pdf]

Table S1. Physico chemical properties of wheat SBT gene family

Genename	GeneID	chromosome	Aminosize(aa)	MW(Da)	PI	Subcellularlocalization
TaSTB1	TraesCS1A02G187300.1	1A	755	79134.31	5.52	extr
TaSTB2	TraesCS1A02G233100.1	1A	731	77181.78	8.25	chlo
TaSTB3	TraesCS1A02G235700.1	1A	626	65345.59	8.16	chlo
TaSTB4	TraesCS1A02G237300.1	1A	724	76810.53	8.56	chlo
TaSTB5	TraesCS1A02G237900.1	1A	759	78760.53	6.5	chlo
TaSTB6	TraesCS1A02G238600.1	1A	738	78080.98	8.56	extr
TaSTB7	TraesCS1A02G272000.1	1A	745	78745.33	5.81	chlo
TaSTB8	TraesCS1A02G292900.1	1A	745	79784.01	5.97	extr
TaSTB9	TraesCS1A02G404600.1	1A	762	81694.33	6.36	extr
TaSTB10	TraesCS2A02G025400.1	2A	779	80876.59	5.92	extr
TaSTB11	TraesCS2A02G037100.1	2A	777	80625.43	6.35	E.R.
TaSTB12	TraesCS2A02G099500.1	2A	747	78439.02	7.94	chlo
TaSTB13	TraesCS2A02G099600.1	2A	744	78219.57	6.83	chlo
TaSTB14	TraesCS2A02G099700.1	2A	358	38144.57	9.18	extr
TaSTB15	TraesCS2A02G100100.1	2A	592	61643.96	9.08	E.R.
TaSTB16	TraesCS2A02G303100.1	2A	769	81071.89	8.2	extr
TaSTB17	TraesCS2A02G390000.1	2A	811	85810.41	6.26	extr
TaSTB18	TraesCS2A02G398900.1	2A	777	81898.83	7.58	extr
TaSTB19	TraesCS2A02G408300.1	2A	747	79027.15	6.47	mito
TaSTB20	TraesCS2A02G408400.1	2A	747	79196.34	6.5	extr
TaSTB21	TraesCS2A02G408600.1	2A	743	78058.45	6.02	extr
TaSTB22	TraesCS2A02G408900.1	2A	801	85293.94	5.54	extr
TaSTB23	TraesCS2A02G444900.1	2A	776	80004.02	6.86	chlo
TaSTB24	TraesCS2A02G504400.1	2A	747	78468.93	5.53	chlo
TaSTB25	TraesCS2A02G504500.1	2A	745	78347.81	5.47	plas
TaSTB26	TraesCS2A02G557800.1	2A	489	51849.89	6.73	chlo
TaSTB27	TraesCS3A02G000100.1	3A	752	80518.71	7.64	chlo
TaSTB28	TraesCS3A02G000200.1	3A	752	80484.7	7.64	cyto
TaSTB29	TraesCS3A02G138200.1	3A	714	76591.49	8.69	chlo
TaSTB30	TraesCS3A02G138300.1	3A	740	78869.77	7.64	chlo
TaSTB31	TraesCS3A02G138400.1	3A	766	82187.6	7.31	chlo
TaSTB32	TraesCS3A02G138600.1	3A	500	53403.19	8.33	chlo
TaSTB33	TraesCS3A02G191500.1	3A	782	81140.75	7.25	E.R.
TaSTB34	TraesCS3A02G254900.1	3A	767	80341.05	6.04	chlo
TaSTB35	TraesCS3A02G283600.2	3A	778	81973.07	8.9	chlo
TaSTB36	TraesCS3A02G284000.1	3A	737	77269.6	8.73	extr
TaSTB37	TraesCS3A02G297600.1	3A	846	89204.78	6.22	extr/vacu
TaSTB38	TraesCS3A02G327300.1	3A	741	79316.35	6.85	chlo
TaSTB39	TraesCS3A02G380200.1	3A	770	79997.32	5.93	extr
TaSTB40	TraesCS3A02G380300.1	3A	763	79757	5.77	extr

TaSTB41	TraesCS3A02G420500.1	3A	777	79705.35	9.05	extr
TaSTB42	TraesCS4A02G031100.1	4A	794	84518.3	6.45	chlo
TaSTB43	TraesCS4A02G071500.1	4A	782	80446.34	8.84	vacu
TaSTB44	TraesCS4A02G237500.1	4A	767	78493.23	5.71	chlo
TaSTB45	TraesCS4A02G407900.1	4A	795	83689.3	7.99	extr
TaSTB46	TraesCS4A02G423900.1	4A	737	77916.73	6.06	chlo
TaSTB47	TraesCS5A02G036000.1	5A	742	79581.47	7.9	extr
TaSTB48	TraesCS5A02G188600.1	5A	768	81634.66	6.55	cyto/E.R.
TaSTB49	TraesCS5A02G196800.1	5A	772	82181.19	8.17	chlo
TaSTB50	TraesCS5A02G196900.1	5A	767	81476.55	8.48	chlo/mito
TaSTB51	TraesCS5A02G214200.1	5A	842	87217.86	9.05	extr
TaSTB52	TraesCS5A02G255900.1	5A	813	86126.62	9.65	plas
TaSTB53	TraesCS5A02G316000.1	5A	785	81467.74	9.38	vacu
TaSTB54	TraesCS5A02G318800.1	5A	766	81594.88	8.83	chlo
TaSTB55	TraesCS5A02G347300.1	5A	718	75944.26	5.98	extr
TaSTB56	TraesCS5A02G399800.1	5A	765	78754.46	7.26	vacu
TaSTB57	TraesCS5A02G520700.1	5A	756	79046.78	7.06	chlo/vacu
TaSTB58	TraesCS6A02G028800.1	6A	748	80029.92	6.64	plas
TaSTB59	TraesCS6A02G036800.1	6A	787	80969.63	8.37	chlo
TaSTB60	TraesCS6A02G112200.1	6A	738	78887.81	6.32	E.R.
TaSTB61	TraesCS6A02G134300.1	6A	423	45241.58	8.51	chlo
TaSTB62	TraesCS6A02G143600.1	6A	715	74560.41	6.83	cyto
TaSTB63	TraesCS6A02G161600.1	6A	786	83240.55	8.1	extr
TaSTB64	TraesCS6A02G173300.1	6A	757	81017.72	6.26	chlo
TaSTB65	TraesCS6A02G245600.1	6A	810	86481.58	6.62	chlo
TaSTB66	TraesCS6A02G246300.2	6A	1364	149260.13	6.17	chlo
TaSTB67	TraesCS6A02G337800.1	6A	786	81327.04	8.63	vacu
TaSTB68	TraesCS6A02G337900.1	6A	742	76723.76	6	E.R.
TaSTB69	TraesCS6A02G339100.1	6A	780	80417.62	8.17	plas
TaSTB70	TraesCS6A02G339200.1	6A	784	80669.2	7.19	chlo
TaSTB71	TraesCS6A02G339400.1	6A	783	80872.52	5.92	chlo
TaSTB72	TraesCS7A02G037500.1	7A	616	64210.44	9.51	chlo
TaSTB73	TraesCS7A02G118100.1	7A	737	78453.29	7.24	extr
TaSTB74	TraesCS7A02G118200.1	7A	682	73288.82	7.59	E.R.
TaSTB75	TraesCS7A02G118300.1	7A	739	79324.65	7.91	chlo
TaSTB76	TraesCS7A02G118400.1	7A	399	42957.99	5.41	chlo
TaSTB77	TraesCS7A02G118500.1	7A	388	41701.61	5.69	chlo
TaSTB78	TraesCS7A02G118600.1	7A	271	28973.17	7.74	chlo
TaSTB79	TraesCS7A02G118700.1	7A	319	34064.69	8.2	mito
TaSTB80	TraesCS7A02G124000.2	7A	1039	115442.4	6.32	chlo
TaSTB81	TraesCS7A02G124600.1	7A	739	78258.54	7.04	chlo
TaSTB82	TraesCS7A02G128100.1	7A	742	78022.29	6.68	chlo

TaSTB83	TraesCS7A02G211600.1	7A	768	79225.3	9.46	E.R.
TaSTB84	TraesCS7A02G381400.1	7A	798	84399.86	7.22	cyto
TaSTB85	TraesCS7A02G421400.1	7A	818	86858.78	7.05	E.R.
TaSTB86	TraesCS7A02G488500.1	7A	803	85176.49	7.61	chlo
TaSTB87	TraesCS1B02G180200.1	1B	850	90248.33	8.85	extr
TaSTB88	TraesCS1B02G195100.1	1B	755	79248.46	5.54	vacu/E.R.
TaSTB89	TraesCS1B02G246100.1	1B	720	76023.4	8.01	cyto
TaSTB90	TraesCS1B02G246400.1	1B	774	80575.6	6.43	vacu
TaSTB91	TraesCS1B02G250400.1	1B	345	36359.81	8.76	mito
TaSTB92	TraesCS1B02G250500.1	1B	737	77595.69	7.63	extr
TaSTB93	TraesCS1B02G281800.1	1B	414	44570.88	9.06	extr
TaSTB94	TraesCS1B02G282000.1	1B	744	78518.76	5.59	cyto
TaSTB95	TraesCS1B02G434100.2	1B	777	83515.75	6.6	cyto
TaSTB96	TraesCS1B02G434200.2	1B	746	79835.6	6.38	vacu
TaSTB97	TraesCS1B02G453400.1	1B	740	78711.86	8.63	extr
TaSTB98	TraesCS2B02G038300.1	2B	678	70656.89	6.16	chlo
TaSTB99	TraesCS2B02G050700.2	2B	700	72421.33	4.89	E.R.
TaSTB100	TraesCS2B02G050800.1	2B	775	80817.85	7.17	extr
TaSTB101	TraesCS2B02G116200.1	2B	754	79611.34	7.62	chlo/extr
TaSTB102	TraesCS2B02G116300.1	2B	746	78216.78	7.62	chlo
TaSTB103	TraesCS2B02G116400.1	2B	335	34742.83	9.65	vacu
TaSTB104	TraesCS2B02G116600.1	2B	744	78186.42	6.99	extr
TaSTB105	TraesCS2B02G117100.1	2B	751	78550.71	7.08	extr
TaSTB106	TraesCS2B02G136200.1	2B	730	77640.58	5.61	chlo
TaSTB107	TraesCS2B02G319700.1	2B	771	81113.74	7.96	chlo
TaSTB108	TraesCS2B02G409200.2	2B	811	85928.45	6.38	chlo
TaSTB109	TraesCS2B02G416900.1	2B	778	82100	6.58	chlo
TaSTB110	TraesCS2B02G426600.1	2B	744	78139.82	5.87	extr
TaSTB111	TraesCS2B02G426700.1	2B	628	65754.53	6.53	chlo/extr
TaSTB112	TraesCS2B02G426800.1	2B	747	78953.03	6.23	chlo
TaSTB113	TraesCS2B02G426900.1	2B	757	79330	6.19	vacu
TaSTB114	TraesCS2B02G427200.1	2B	801	85313.07	5.5	vacu
TaSTB115	TraesCS2B02G532700.1	2B	736	77438.75	5.46	chlo
TaSTB116	TraesCS2B02G532800.1	2B	747	78223.69	5.81	vacu
TaSTB117	TraesCS3B02G004100.1	3B	751	80425.75	7.9	extr
TaSTB118	TraesCS3B02G004200.1	3B	754	80981.94	7.08	extr/vacu
TaSTB119	TraesCS3B02G004300.1	3B	309	33668.7	8.74	chlo
TaSTB120	TraesCS3B02G004600.1	3B	797	85202.81	7.28	chlo
TaSTB121	TraesCS3B02G155900.1	3B	758	81171.93	7.3	chlo
TaSTB122	TraesCS3B02G156000.1	3B	584	62517.87	8.53	chlo
TaSTB123	TraesCS3B02G156100.1	3B	562	59803.71	8.44	chlo
TaSTB124	TraesCS3B02G156200.1	3B	392	41949.64	6.89	nucl

TaSTB125	TraesCS3B02G219500.3	3B	787	81536.21	6.96	E.R.
TaSTB126	TraesCS3B02G286900.1	3B	780	83675.24	7.55	extr
TaSTB127	TraesCS3B02G317300.1	3B	779	81753.79	8.91	extr
TaSTB128	TraesCS3B02G339100.1	3B	844	88706.36	6.22	chlo
TaSTB129	TraesCS3B02G356700.1	3B	747	80236.44	6.62	plas
TaSTB130	TraesCS3B02G412800.1	3B	603	62146.48	6.5	vacu
TaSTB131	TraesCS3B02G412900.1	3B	764	79868.21	5.72	E.R.
TaSTB132	TraesCS3B02G455900.1	3B	445	46320.61	9.41	E.R.
TaSTB133	TraesCS4B02G022600.1	4B	783	83750.93	6.03	vacu
TaSTB134	TraesCS4B02G077600.1	4B	767	78391.15	5.99	chlo
TaSTB135	TraesCS4B02G085500.1	4B	482	51633.44	5.89	vacu
TaSTB136	TraesCS4B02G227400.1	4B	782	80473.27	8.53	extr
TaSTB137	TraesCS4B02G274900.2	4B	791	84240.99	6.4	chlo
TaSTB138	TraesCS4B02G307400.1	4B	795	83816.6	8.74	extr
TaSTB139	TraesCS4B02G352100.1	4B	756	79163.97	7.63	chlo
TaSTB140	TraesCS5B02G024600.1	5B	772	82475.11	7.87	chlo
TaSTB141	TraesCS5B02G036600.2	5B	485	52432.07	6.52	extr
TaSTB142	TraesCS5B02G186700.1	5B	795	84620.12	8.8	chlo
TaSTB143	TraesCS5B02G186800.1	5B	221	24221.41	8.45	extr
TaSTB144	TraesCS5B02G187000.1	5B	566	59664.26	9.48	chlo
TaSTB145	TraesCS5B02G209500.1	5B	773	79757.2	7.94	vacu
TaSTB146	TraesCS5B02G255200.1	5B	810	86107.33	9.09	chlo
TaSTB147	TraesCS5B02G319300.2	5B	776	82857.32	8.67	extr/vacu
TaSTB148	TraesCS5B02G367400.1	5B	741	79249.05	6.34	chlo
TaSTB149	TraesCS5B02G368100.1	5B	739	78797.03	6.02	chlo
TaSTB150	TraesCS5B02G404600.1	5B	764	78604.33	7	plas
TaSTB151	TraesCS6B02G040900.1	6B	752	79539.89	6.18	extr
TaSTB152	TraesCS6B02G041200.1	6B	757	81141.39	7.68	extr
TaSTB153	TraesCS6B02G051400.1	6B	789	81729.62	8.89	vacu
TaSTB154	TraesCS6B02G171900.1	6B	731	75732.25	5.79	extr
TaSTB155	TraesCS6B02G201000.1	6B	765	81882.44	6.4	extr
TaSTB156	TraesCS6B02G278200.1	6B	1361	148862.8	6.27	chlo
TaSTB157	TraesCS6B02G278800.1	6B	846	90401.07	7.65	extr
TaSTB158	TraesCS6B02G368500.1	6B	786	81511.45	9.12	extr/vacu
TaSTB159	TraesCS6B02G368600.1	6B	785	81614.34	6.97	vacu
TaSTB160	TraesCS6B02G369400.1	6B	779	80756.59	8.75	vacu
TaSTB161	TraesCS6B02G370000.1	6B	829	85463.47	8.16	extr/vacu
TaSTB162	TraesCS6B02G370100.1	6B	784	81035.64	7.57	nucl
TaSTB163	TraesCS6B02G370300.1	6B	780	80747.29	6.03	vacu
TaSTB164	TraesCS7B02G015100.1	7B	737	78197.6	6.35	chlo
TaSTB165	TraesCS7B02G015200.1	7B	740	79152.39	6.81	chlo
TaSTB166	TraesCS7B02G015300.1	7B	729	78263.15	6.35	chlo/mito

TaSTB167	TraesCS7B02G022900.2	7B	1038	115338.19	6.07	E.R.
TaSTB168	TraesCS7B02G027900.1	7B	406	42211.3	5.35	vacu
TaSTB169	TraesCS7B02G043900.1	7B	690	71830.14	6.99	chlo
TaSTB170	TraesCS7B02G118600.1	7B	768	79172.19	9.41	chlo
TaSTB171	TraesCS7B02G283300.1	7B	798	84937.75	8.76	chlo
TaSTB172	TraesCS7B02G321700.1	7B	850	90436.79	6.84	chlo
TaSTB173	TraesCS7B02G391700.1	7B	555	58706.61	8.6	extr
TaSTB174	TraesCS7B02G452500.1	7B	781	80459.88	8.93	extr
TaSTB175	TraesCS1D02G189400.1	1D	755	79306.5	5.43	plas
TaSTB176	TraesCS1D02G234200.1	1D	738	77885.51	8.42	chlo
TaSTB177	TraesCS1D02G234500.1	1D	701	74223.41	6.28	chlo
TaSTB178	TraesCS1D02G234700.1	1D	756	78382.96	6.06	chlo
TaSTB179	TraesCS1D02G238800.1	1D	737	77609.58	7.67	vacu
TaSTB180	TraesCS1D02G272900.1	1D	745	78704.06	5.73	E.R.
TaSTB181	TraesCS1D02G412300.1	1D	762	81968.57	6.39	extr
TaSTB182	TraesCS1D02G412400.3	1D	751	80454.2	6.36	vacu
TaSTB183	TraesCS1D02G430100.1	1D	431	45800.36	8.8	extr
TaSTB184	TraesCS2D02G027400.1	2D	767	79786.34	5.91	vacu
TaSTB185	TraesCS2D02G029200.1	2D	821	86105.03	6.77	mito
TaSTB186	TraesCS2D02G036400.1	2D	777	80475.25	6.38	chlo
TaSTB187	TraesCS2D02G036500.1	2D	801	83525.95	6.53	plas
TaSTB188	TraesCS2D02G098800.1	2D	360	37362.49	9.48	plas
TaSTB189	TraesCS2D02G098900.1	2D	320	33577.78	6.3	chlo
TaSTB190	TraesCS2D02G099000.1	2D	745	78279.02	8.54	vacu
TaSTB191	TraesCS2D02G099100.1	2D	741	77883.02	7.02	plas
TaSTB192	TraesCS2D02G099200.1	2D	609	64136.96	6.52	chlo
TaSTB193	TraesCS2D02G099600.1	2D	751	78535.77	7.65	chlo
TaSTB194	TraesCS2D02G301700.1	2D	771	80958.66	7.96	plas
TaSTB195	TraesCS2D02G388700.2	2D	827	87652.48	6.05	chlo
TaSTB196	TraesCS2D02G396400.1	2D	777	82149.12	7.02	cyto
TaSTB197	TraesCS2D02G406000.1	2D	801	85373.9	5.44	plas
TaSTB198	TraesCS2D02G406300.1	2D	757	79133.78	6.65	chlo
TaSTB199	TraesCS2D02G406400.1	2D	748	79226.48	6.44	E.R.
TaSTB200	TraesCS2D02G406500.1	2D	749	78927.72	6.09	cyto
TaSTB201	TraesCS2D02G406600.1	2D	708	75349.82	5.98	chlo
TaSTB202	TraesCS2D02G505000.1	2D	745	78348.19	6.23	vacu
TaSTB203	TraesCS2D02G505100.1	2D	749	78397.74	5.6	plas
TaSTB204	TraesCS3D02G008600.1	3D	795	84997.27	6.87	plas
TaSTB205	TraesCS3D02G008700.1	3D	752	80389.4	6.8	plas
TaSTB206	TraesCS3D02G138900.1	3D	681	72828.97	6.39	nucl
TaSTB207	TraesCS3D02G194100.1	3D	730	75334.33	8.18	chlo
TaSTB208	TraesCS3D02G255800.1	3D	751	79153.78	6.57	vacu

TaSTB209	TraesCS3D02G283500.1	3D	778	81726.92	9.1	plas
TaSTB210	TraesCS3D02G283900.1	3D	758	79977.5	9.43	chlo
TaSTB211	TraesCS3D02G304700.1	3D	845	89343.99	6.42	chlo
TaSTB212	TraesCS3D02G320700.1	3D	741	79274.33	6.72	chlo
TaSTB213	TraesCS3D02G373400.1	3D	765	79759.04	5.71	chlo
TaSTB214	TraesCS3D02G416100.1	3D	779	80028.77	9.08	extr
TaSTB215	TraesCS4D02G076000.1	4D	764	78167.93	5.99	plas
TaSTB216	TraesCS4D02G228200.1	4D	785	80718.52	8.75	plas
TaSTB217	TraesCS4D02G273500.3	4D	795	84620.46	6.29	E.R.
TaSTB218	TraesCS4D02G305500.1	4D	797	83901.69	8.74	chlo
TaSTB219	TraesCS5D02G033200.1	5D	748	80014.04	7.32	extr
TaSTB220	TraesCS5D02G044100.2	5D	757	81095.12	8.31	chlo
TaSTB221	TraesCS5D02G194300.1	5D	709	75762.18	8.19	plas
TaSTB222	TraesCS5D02G194400.1	5D	758	80433.69	9.06	plas
TaSTB223	TraesCS5D02G194600.1	5D	763	81220.46	8.95	chlo
TaSTB224	TraesCS5D02G202400.1	5D	769	82079.54	7.94	chlo
TaSTB225	TraesCS5D02G217700.1	5D	772	79463.65	7.97	plas
TaSTB226	TraesCS5D02G264000.1	5D	811	86231.62	9.54	chlo
TaSTB227	TraesCS5D02G322200.1	5D	784	81468.8	9.41	vacu
TaSTB228	TraesCS5D02G325000.1	5D	773	82365.79	8.67	extr
TaSTB229	TraesCS5D02G409600.1	5D	764	78611.32	6.81	nucl/cyto
TaSTB230	TraesCS5D02G554200.1	5D	227	24402.5	5.12	extr
TaSTB231	TraesCS6D02G033000.1	6D	759	81023.35	8.57	chlo
TaSTB232	TraesCS6D02G101200.1	6D	743	79632.73	6.66	chlo/E.R.
TaSTB233	TraesCS6D02G162400.1	6D	735	78835.2	6.66	chlo/vacu
TaSTB234	TraesCS6D02G228000.1	6D	785	83883.59	6.43	chlo
TaSTB235	TraesCS6D02G228700.4	6D	1491	163207.69	7.06	cyto
TaSTB236	TraesCS6D02G318700.1	6D	666	69065.9	8.85	chlo
TaSTB237	TraesCS6D02G319700.1	6D	788	81770.52	6.18	extr/vacu
TaSTB238	TraesCS6D02G319800.1	6D	784	81181.76	6.9	vacu
TaSTB239	TraesCS6D02G319900.2	6D	834	85751.82	8.64	vacu
TaSTB240	TraesCS7D02G113600.1	7D	589	62370.04	7.27	chlo
TaSTB241	TraesCS7D02G113700.1	7D	651	69409.27	6.7	cyto
TaSTB242	TraesCS7D02G113800.1	7D	737	78291.8	6.29	cyto
TaSTB243	TraesCS7D02G113900.1	7D	730	78096.24	7.26	vacu
TaSTB244	TraesCS7D02G114000.1	7D	583	61890.23	5.92	chlo
TaSTB245	TraesCS7D02G121900.1	7D	1113	123604.03	7.98	chlo
TaSTB246	TraesCS7D02G126900.1	7D	376	39591.4	5.73	E.R.
TaSTB247	TraesCS7D02G213300.1	7D	768	79342.49	9.45	cyto
TaSTB248	TraesCS7D02G377700.1	7D	808	85804.62	7.87	extr
TaSTB249	TraesCS7D02G413700.1	7D	819	87084.14	7.66	extr
TaSTB250	TraesCS7D02G475000.1	7D	755	79632.25	6.29	chlo

TaSTB251	TraesCSU02G001100.1	U0	751	80656.36	6.29	cyto/extr
TaSTB252	TraesCSU02G025500.1	U0	777	79918.85	6.88	vacu
TaSTB253	TraesCSU02G039600.1	U0	220	24294.93	8.87	cyto
TaSTB254	TraesCSU02G079100.1	U0	739	78093.07	5.96	chlo
TaSTB255	TraesCSU02G208100.1	U0	749	76064.54	7.11	vacu
