# Supplementary material for: Genome-Wide Identification, Phylogeny and Expression Analysis of Subtilisin (SBT) Gene Family under Wheat Biotic and Abiotic Stress
Source: Plants (Basel). 2023 Aug 25;12(17):3065. doi: 10.3390/plants12173065 (PMC10489890; doi:10.3390/plants12173065)
Supplement: Supplementary file 1 [file plants-12-03065-s001.zip › Table S3.pdf]

Table S3. Primers for TaSBT7, 26, 102, 111, 193, 213 gene cloning.

Primer name	Primer sequence（5’-3’）
TaSBT7-F	GAGGAGCTCGACAGGCTC
TaSBT7-R	CGTTCTCACCGTAGTTGGAC
TaSBT26-F	ATACTCGCCGCCATGGAC
TaSBT26-R	CAGCCGAAGTTGACACGAAG
TaSBT102-F	AACAATGGGCCTAGACCGC
TaSBT102-R	CCGGACGAGTTCTTGGCTT
TaSBT111-F	TCATGGGACAAACGGTAACAC
TaSBT111-R	CGATTTTGCCTCGCACCTT
TaSBT193-F	CGTGTTGTCGATGTCCTTTG
TaSBT193-R	GCAACGGTGATAGCCCAC
TaSBT213-F	CCGGAGATTCCACCGCAT
TaSBT213-R	TATCTTGTTCGCGCCGAGC
